# Simultaneous Quantification and Pharmacokinetic Characterization of Doxapram and 2-Ketodoxapram in Porcine Plasma and Brain Tissue

**DOI:** 10.3390/pharmaceutics14040762

**Published:** 2022-03-31

**Authors:** Manuel Kraft, Kathrin I. Foerster, Felix Wiedmann, Max Sauter, Amelie Paasche, Pablo L. Blochberger, Baran Yesilgöz, Yannick L’hoste, Norbert Frey, Walter E. Haefeli, Jürgen Burhenne, Constanze Schmidt

**Affiliations:** 1 Department of Cardiology, Heidelberg University Hospital, 69120 Heidelberg, Germany; manuel.kraft@med.uni-heidelberg.de (M.K.); felix.wiedmann@med.uni-heidelberg.de (F.W.); ameliecolleen.paasche@med.uni-heidelberg.de (A.P.); pabloleon.blochberger@med.uni-heidelberg.de (P.L.B.); baran.yesilgoez@med.uni-heidelberg.de (B.Y.); y.lhoste@gmail.com (Y.L.); norbert.frey@med.uni-heidelberg.de (N.F.); 2 DZHK (German Centre for Cardiovascular Research), Partner Site Heidelberg/Mannheim, Heidelberg University Hospital, 69120 Heidelberg, Germany; 3 HCR, Heidelberg Centre for Heart Rhythm Disorders, Heidelberg University Hospital, 69120 Heidelberg, Germany; 4 Department of Clinical Pharmacology and Pharmacoepidemiology, Heidelberg University Hospital, 69120 Heidelberg, Germany; kathrin.foerster@med.uni-heidelberg.de (K.I.F.); max.sauter@med.uni-heidelberg.de (M.S.); walter-emil.haefeli@med.uni-heidelberg.de (W.E.H.); juergen.burhenne@med.uni-heidelberg.de (J.B.)

**Keywords:** doxapram, 2-ketodoxapram, UPLC-MS/MS, atrial fibrillation, pharmacokinetics, central nervous system, TASK-1, *KCNK3*, protein binding

## Abstract

Atrial fibrillation (AF) is an arrhythmia associated with an increased stroke risk and mortality rate. Current treatment options leave unmet needs in AF therapy. Recently, doxapram has been introduced as a possible new option for AF treatment in a porcine animal model. To better understand its pharmacokinetics, three German Landrace pigs were treated with intravenous doxapram (1 mg/kg). Plasma and brain tissue samples were collected. For the analysis of these samples, an ultra performance liquid chromatography tandem mass spectrometry (UPLC-MS/MS) assay for the simultaneous measurement of doxapram and its active metabolite 2-ketodoxapram was developed and validated. The assay had a lower limit of quantification (LLOQ) of 10 pg/mL for plasma and 1 pg/sample for brain tissue. In pigs, doxapram pharmacokinetics were biphasic with a terminal elimination half-life (t_1/2_) of 1.38 ± 0.22 h and a maximal plasma concentration (c_max_) of 1780 ± 275 ng/mL. Its active metabolite 2-ketodoxapram had a t_1/2_ of 2.42 ± 0.04 h and c_max_ of 32.3 ± 5.5 h after administration of doxapram. Protein binding was 95.5 ± 0.9% for doxapram and 98.4 ± 0.3% for 2-ketodoxapram with a brain-to-plasma ratio of 0.58 ± 0.24 for doxapram and 0.12 ± 0.02 for 2-ketodoxapram. In conclusion, the developed assay was successfully applied to the creation of pharmacokinetic data for doxapram, possibly improving the safety of its usage.

## 1. Introduction

Atrial fibrillation (AF), the most common sustained arrhythmia, is associated with an increased rate of stroke and mortality [1]. However, while oral anticoagulants can significantly lower the risk of ischemic strokes, AF patients still suffer from a higher mortality rate compared to the sinus rhythm (SR) population [2]. Current treatment options have an insufficient efficacy and/or are connected with cardiac and extracardiac side effects, emphasizing an unmet need in AF therapy [3]. To fill this gap, it has long been speculated that atrial-specifically expressed ion channels would be ideal targets for the treatment of AF [3]. 

In recent years, the two-pore domain potassium channel TASK-1 has been identified as such a target structure that is atrial specific. Furthermore, its expression is upregulated in AF patients, emphasizing its important role in shaping the atrial action potential in these patients [4]. In addition, it was shown that pharmacological inhibition of TASK-1 facilitated the cardioversion of AF to SR in a porcine AF model [5,6,7]. This model has proven to be very relevant and reliable as the cardiovascular system in pigs is very similar to humans. Especially important for our experiments, the expression of TASK-1 is regulated in a similar way in both species [4,8] Therefore, data from this porcine AF model can be used as a basis for first in human trails. 

Doxapram, a well-established respiratory stimulant, has a strong inhibitory effect on TASK-1, leading to cardioversion of AF in pigs [5]. Some known adverse events of doxapram use are hypertension, dyspnoea, coughing, tachypnoea, headache, dizziness, flushing, sweating, perineal warmth, tremor, nausea, vomiting, diarrhoea, urinary retention, and muscle spasticity. Furthermore, there is conflicting information about the proconvulsant potential of doxapram [9,10]. More serious adverse events observed for doxapram are based on its effect on the central nervous system. These have been primarily described in case reports and include psychosis with hallucinations, severe and violent restlessness, and confusion [10]. To establish a concentration–response relationship of doxapram in pigs, its pharmacokinetics and pharmacodynamics need to be characterized. While much is known about the pharmacodynamics in pigs, little information is available on its kinetics [5]. 

To establish doxapram pharmacokinetics in pigs, we developed an ultra performance liquid chromatography tandem mass spectrometry (UPLC-MS/MS) assay for the simultaneous measurement of doxapram and its active metabolite 2-ketodoxapram in porcine plasma. In a second step, and to assess the probability of adverse events within the central nervous system, the assay was adapted to the quantification of both analytes in brain tissue. Therefore, we describe in this manuscript the development, validation, and application of these assays and present first pharmacokinetic data of doxapram and 2-ketodoxapram in pigs. Furthermore, information on the protein binding and the permeability across the blood–brain barrier is provided. 

## 2. Materials and Methods

### 2.1. Animal Study and Sample Generation

The study protocol was authorized by the responsible local animal welfare committee (Regierungspräsidium Karlsruhe, Germany, reference numbers G198-20), and the experiments were performed according to EU Directive 2010/63/EU and the German Law on the Protection of Animals. 

For drug injection and blood sampling, three German Landrace pigs (bodyweight: 35–40 kg) were anaesthetised, and central vein catheters were implanted. The following experiments were performed on awake animals unless stated otherwise. For 14 d, starting on day 2 after the operation, daily intravenous (i.v.) fast bolus injections (within 1 min) of 1 mg/kg doxapram (Dopram^®^, Carinopharm, Elze, Germany) were administered to all three animals. Before drug administration, blood samples were taken in Lithium-Heparin tubes (Sarstedt, Nürmbrecht, Germany). At day 2, the first day of drug administration, additional samples were collected at timepoints of 5 min, 10 min, 15 min, 20 min, 30 min, 45 min, 60 min, 2 h, 3 h, 4 h, 5 h, 6 h, 7 h, 8 h, and 9 h after injection. The samples were centrifuged at 2500× *g* for 10 min. The plasma was transferred to collection tubes and stored at −20 °C until analysis. 

At the end of the 14-day period, the three pigs were euthanised, under deep anaesthesia (2 mg/kg propofol i.v., Propofol 2% MCT Fresenius, Fresenius Kabi, Bad Homburg, Germany) and strong pain medication (0.02 mg/kg buprenorphine i.v., Buprenovet^®^, Bayer Vital, Leverkusen, Germany), by an administration of potassium chloride (40 mmol, Kaliumchlorid 7,45% gefärbt, B Braun Melsungen, Melsungen, Germany) directly into the heart. Afterwards, a final blood sample was taken and prepared as described. At the same time, brain tissue samples from both hemispheres were collected and immediately deep frozen in liquid nitrogen and stored at −80 °C until analysis. 

### 2.2. Reagents and Solvents 

Doxapram (C_24_H_30_N_2_O_2_, 97.7%, 378.2 g/mol) for method validation was bought from Biozol Diagnostika Vertrieb (Eching, Germany). 2-Ketodoxapram (C_24_H_28_N_2_O_3,_ 98.8%; 392.2 g/mol); the stable isotopically labelled internal standards (IS) doxapram-d5 (99.6%, 383.2 g/mol) and 2-ketodoxapram-d5 (99.6%, 397.2 g/mol) were synthesised by TLC Pharmaceutical Standards (Newmarket, ON, Canada). 

Acetonitrile (ACN) and formic acid (FA) were purchased from Biosolve (ULC/MS grade; Valkenswaard, The Netherlands), and tert-butyl methyl ether (TBME), boric acid, sodium hydroxide, and hydrochloric acid were purchased from Merck (Darmstadt, Germany). Ultrapure water was freshly prepared with an arium^®^ mini system (Sartorius, Göttingen, Germany). Analyte-free porcine plasma and brain tissue for assay validation was available from untreated control animals from previously performed studies. 

### 2.3. Preparation of Standard Solutions

For each analyte and IS, separate stock solutions were prepared by independently weighing and dissolving them in ACN/water (1/1, *v*/*v*). These stocks were mixed and diluted with ACN/water to produce eight calibrators, four quality controls (QC), one lower limit of quantification (LLOQ), and one IS solution for the three different calibration ranges, covering 10–10,000 pg/mL (low plasma concentrations; LLOQ: 10 pg/mL), 1–2500 ng/mL (high plasma concentrations; LLOQ: 1 ng/mL), and 1–2500 pg/sample (brain tissue; LLOQ: 1 pg/sample). A fifth QC solution, with a concentration higher than the calibration range, was prepared to monitor the integrity of sample dilution. The QC and LLOQ solutions were produced from stock solutions separately weighted from the stock solution used for the preparation of the calibrators. The stock solutions were stored at −20 °C; the calibrator, QC, and IS working solutions were stored at 4 °C. 

### 2.4. Sample Preparation

For QC and calibrator samples, the respective working solutions (25 µL) were mixed with blank plasma (100 µL) or blank tissue matrix (100 µL; 40 mg/mL brain tissue in water), whereas, for analytical samples 100 µL of plasma or 100 µL of brain solution (40 mg/mL brain tissue in water) was used, and 25 µL of ACN/water (1/1, *v*/*v*) was added for volume compensation. All samples except blanks were mixed with 25 µL of IS working solution. For the purpose of liquid–liquid extraction, 100 µL of 0.2 M borate buffer (pH 9) and 2 mL TBME were added to each sample. After 10 min of overhead shaking and subsequent centrifugation (10 min, 3000× *g*, 15 °C), 25 µL (high concentration assay) or 1.5 mL (low concentration assay, brain tissue assay) of the organic phase was transferred to a glass tube and evaporated to dryness under a heated stream of nitrogen (10 min, 40 °C). The residue was dissolved in 500 µL (high concentration assay, brain tissue assay) or 100 µL (low concentration assay) eluent (water with 9.75% ACN and 0.1% FA) in an ultrasonic device for 1 min and transferred to a 96-well collection plate (Waters Corporation, Milford, MA, USA) for measurement. 

### 2.5. Instrumental Analysis

An Acquity UPLC^®^ I-class system connected to a Xevo TQ-S tandem mass spectrometer (Waters) was used for analysis. The samples were kept at 15 °C in the autosampler. 

Chromatic separation was achieved on an Acquity UPLC^®^ BEH C18 column (1.7 µm, 2.1 × 50 mm; Waters) heated to 40 °C in gradient mode with a flow rate of 0.5 mL/min and an injection volume of 10 µL. As eluents, a mixture of water, 5% ACN and 0.1% FA (A), and of ACN with 0.1% FA (B) was used. The gradient started with 95% A/5% B for 0.5 min, changed to 5% A/95% B until 3.5 min, and reversed back to 95% A/5% B until 4.0 min. Subsequently, a second change to 5% A/95% B was implemented until 5.0 min to clean the column of any residues, ending with a change back to 95% A/5% B until 5.5 min and holding this until the end of the 6-min run.

After positive ionization with heated electrospray ionization (ESI, Z-spray), mass-to-charge transitions of *m*/*z* 379.5 > 292.3 (doxapram) and *m*/*z* 384.5 > 297.3 (doxapram-d5) were monitored for quantification of doxapram using the multiple reaction monitoring (MRM) mode. For the metabolite, transitions of *m/z* 393.4 > 214.3 (2-ketodoxapram) and *m*/*z* 398.4 > 219.3 (2-ketodoxapram-d5) were monitored. The following instrumental parameters were used: capillary voltage 2 kV, cone voltage 44 V, source temperature 150 °C, cone gas flow (N_2_) 150 L/h, desolvation gas flow (N_2_) 1000 L/h, desolvation temperature 600 °C, collision gas flow (Ar) 0.15 mL/min, and collision energy of 18 V for doxapram/doxapram-d5 and of 23 V for 2-ketodoxapram/2-ketodoxapram-d5. 

### 2.6. Method Validation

The assays used for the quantification of doxapram and its metabolite 2-ketodoxapram in brain tissue and plasma were validated according to the pertinent FDA [11] and EMA [12] guidelines on bioanalytical method validation. To determine accuracy and precision for every assay, the four QC working solutions and the LLOQ were measured six-fold within three analytical runs. The parameters were evaluated within (intra) and between (inter) runs respectively days. The dilution QC samples were diluted 10-fold (plasma) or 100-fold (brain tissue) with blank matrix before addition of IS and measured six-fold to assess dilution integrity. For selectivity determination, six different blank matrix samples were measured. Blank eluent samples were injected directly after the analysis of the highest calibrator of each assay to quantify a possible carry-over effect. Recovery and matrix effect were measured at every QC concentration in triplicates using six different lots of blank matrices. For quantification of recovery, the ratios between drug and respective IS peak areas in samples spiked with QC and IS solutions before sample preparation and afterwards were compared. To assess matrix effects, ratios of peak areas in blank samples spiked with QC and IS solutions after sample preparation were compared to peak area ratios of pure spiking solutions mixed with eluent [13]. 

Autosampler stability was assessed by repeatedly measuring the same QC samples, stored in the autosampler at 15 °C, on two consecutive days. For long-term stability, QC solutions without IS were transferred into blank matrix and stored at −20 °C for at least 14 d before analysis. These samples were compared with QCs produced from freshly prepared stock solutions. The same procedure was used to assess bench-top stability with samples stored at room temperature for 24 h. To quantify the effect of repeated freeze-and-thaw cycles on the analytes, QC samples without IS were frozen for 12 h and subsequently thawed. This was repeated three times before measuring the samples. 

After initial measurement of the study samples, an incurred sample reanalysis of 10% of all samples was performed to verify the reliability of the results. These samples were processed separately from the original measurement run. 

### 2.7. Protein Binding

To quantify the protein binding of doxapram and 2-ketodoxapram, the Rapid Equilibrium Dialysis (RED) device (Thermo Fisher Scientific, Waltham, MA, USA) was used according to the manufacturer’s instructions. Briefly, 300 µL of plasma and 550 µL Dulbecco’s Phosphate Buffered Saline (DPBS; Sigma-Aldrich, St. Louis, MO, USA) were filled in their respective chambers and incubated for 4 h at 37 °C under slow shaking. Subsequently, 100 µL of plasma or DPBS were used for sample preparation and quantification. 

### 2.8. Calculations

Standard calculations were performed with Microsoft Office Excel 2019 (Microsoft Corporation, Redmond, WA, USA) and GraphPad Prism (V9.3.1; GraphPad Software, San Diego, CA, USA). Linear calibration curves with a weighted fitting (1/x^2^) were calculated from the ratio of the peak area of the analyte to the IS separately for both substances, using the Software TargetLynx (V4.1; Waters). The calculation of pharmacokinetic parameters was done using Kinetica (V5.0; Thermo Fisher Scientific). 

## 3. Results and Discussion

### 3.1. Mass Spectrometry and Chromatography

**Figure 1:**
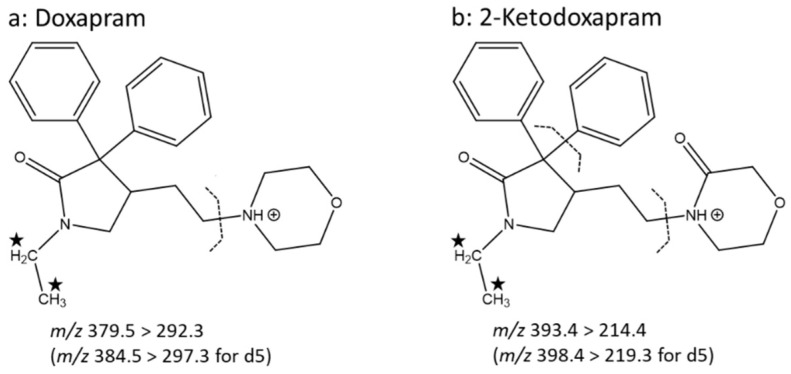
Structures of the analytes doxapram (**a**) and 2-ketodoxapram (**b**). [M + H]^+^ precursor molecules and their fragmentation sites (dashed lines) after electrospray ionization (ESI) in positive ion mode followed by collision-induced decomposition are shown. For doxapram, a collision energy of 18 V was utilized, and for 2-ketodoxapram a collision energy of 23 V was utilized. The five hydrogen atoms marked with an asterisk were exchanged for deuterium atoms in the stable isotopically labelled internal standards (IS).

Under the slightly acidic conditions utilized in the assay, the amine function in the morpholine heterocycle of doxapram was protonated, generating an abundant [M + H]^+^ signal at *m*/*z* 379.5 (*m*/*z* 384.5 for doxapram-d5) with heated ESI in positive ion mode (Figure S1a). The chosen collision conditions yielded a base peak at *m*/*z* 97.3; however, the signal at *m*/*z* 292.3 (*m*/*z* 297.3 for doxapram-d5) showed a better performance during the method validation. Therefore, the corresponding mass transition of *m*/*z* 379.5 to 292.3 was used for the quantification of doxapram (*m*/*z* 384.5 > 297.3 for doxapram-d5) (Figure 1a). For 2-ketodoxapram, a [M + H]^+^ signal at *m*/*z* 393.4 (*m*/*z* 398.4 for 2-ketodoxapram-d5) and base peak at *m*/*z* 214.3 (*m/z* 219.3 for 2-ketodoxapram-d5) were detected (Figure S1b). During method validation, the mass transition of *m*/*z* 393.4 to 214.4 (*m*/*z* 398.4 > 219.3 for 2-ketodoxapram-d5) showed the best performance and was used for quantification (Figure 1b).

Doxapram and 2-ketodoxapram were well separated on a C18 column (Waters BEH C18 UPLC) and kept at 40 °C (Figures S2–S4). A gradient mode with an initial low ACN fraction of 5%, which increased to 95%, clearly separated both analytes, and sharp peaks of 6 s width at baseline were achieved. The retention time of doxapram was 1.61 min and 2.18 min for 2-ketodoxapram, indicating the increase of the lipophilic character of 2-ketodoxapram. 

### 3.2. Sample Preparation

The isolation of doxapram and 2-ketodoxapram from plasma and brain tissue was achieved using liquid–liquid extraction with TBME in slightly alkaline conditions (pH 9.0). After measuring the first samples, it became apparent that the concentrations of doxapram and 2-ketodoxaparam in the porcine samples covered a wide range of concentrations, exceeding the detector’s linear range. To ensure reliable quantification of all samples, two calibration ranges with one for low plasma levels (10 pg/mL–10000 pg/mL) and one for high plasma levels (1 ng/mL–2500 ng/mL) were established and validated. The two assays only differed in the volume taken for analysis. For samples with high concentrations, 25 µL of the TBME phase was transferred for evaporation and afterwards dissolved in 500 µL of eluent. For samples with low concentrations, 1.5 mL of the TBME phase was used and reconstitution was done in only 100 µL of eluent. The brain samples covered a much narrower range. Therefore, a single calibration curve (1 pg/sample–2500 pg/sample) consisting of the transfer of 1.5 mL TBME and dissolving in 500 µL of eluent was sufficient. 

The recovery rate for all assays was satisfactory (Tables S1–S3). The results show a good consistency and reproducibility across all tested QC solutions and assays, passing the requirements set by the EMA and FDA guidance [11,12]. The same applies for possible matrix effects. Neither ion depression nor enhancement was observed for the IS-normalized matrix effects (Tables S1–S3). 

### 3.3. Method Validation

The herein described method fulfils the requirements of the FDA and EMA for bioanalytical methods [11,12]. For all calibration ranges, sample matrices, and analytes linear regression curves with good correlation coefficients (r^2^) ≥ 0.998 were present. The accuracy and precision values were within the limits set by the guidelines (Tables S4–S6). The dilution integrity was verified through a 10-fold (plasma) or 100-fold (brain tissue) dilution with blank matrix and showed accuracies and precision values within the limits (accuracy: 93.4–107.1%; maximal deviation of precision: 2.4%). For testing of selectivity, six different blank brain tissue and plasma matrix samples from control pigs without treatment were used, and no interfering peaks were detected. Blank eluent samples injected directly after the highest calibrators showed no interfering carry-over effect in any of the calibration ranges. 

After repeated measurements of samples stored overnight in the autosampler, the accuracies for all concentrations, matrices, and analytes were within the limits (Tables S7–S9), which is in agreement with findings of Suzuki et al. [14] who showed autosampler stability for 2-ketodoxapram and doxapram over a period of at least 48 h at 10 °C and Flint et al. [15] over a period of 48 h at 15 °C for 2-ketodoxapram and up to 120 h for doxapram. Furthermore, after three freeze-and-thaw cycles, the analytes were stable (Tables S7–S9). The same has been observed for human serum [14]. 

After storage at −20 °C for 16 d, plasma samples were stable (Tables S7 and S8). For longer storage periods at the condition of −20 °C, Suzuki et al. [14] found no significant degradation in human serum after 4 weeks, and Komatsu et al. [16] detected no stability problems in human plasma after 2 months. Furthermore, testing of bench-top stability of plasma samples showed the stability of doxapram and 2-ketodoxapram over this period (Tables S7 and S8). While there are no long-term data available on the stability at room temperature, Suzuki et al. [14] confirmed stability at room temperature for 4 h and at 4 °C for 4 weeks in human serum. Long-term and bench-top stability testing of brain tissue samples was not performed but can be assumed to match stability data in plasma. In summary, the stability data show that long-term storage of samples is possible for at least 2 months and that the sample processing at room temperature followed by storage in the autosampler during the quantification procedure does not cause sample degradation. 

The incurred sample reanalysis for both plasma concentration ranges as well as brain tissue were well within the required limit (Tables S10–S12). In the high concentration range, it was 96.4% (27 of 28 samples); in the low concentration range it was 100% for 2-ketodoxapram (14 of 14 samples), and 92.3% for doxapram (12 of 13 samples), and both tested brain tissue samples were inside the limit.

In the past 40 years, various methods have been published for the quantification of doxapram and 2-ketodoxapram in plasma and serum, using a wide range of techniques and equipment (for an overview see Table 1). To the best of our knowledge, the herein described method is the first fully validated method for the quantification of these analytes in tissue of any kind. Furthermore, even for the quantification of plasma and serum samples, the herein developed method is the only one fully validated by the current FDA and EMA guidelines. While validation data are not available for most methods, Flint et al. [15] performed a validation according to an old FDA guideline from 2003, and Suzuki et al. [14] did not provide any information on the guidelines used. 

Furthermore, our described method has by far the lowest LLOQ with 10 pg/mL, which allows the observation of plasma concentrations over a longer follow-up period and after the administration of smaller drug doses and the quantification of the protein binding. Because doxapram and 2-ketodoxapram are highly bound to proteins (see Section 3.5 for detailed results), the free fraction is very small and requires either a big sample volume or a low LLOQ for exact quantification. In addition, the developed method is the only one using stable isotopically labelled analytes as IS. 

### 3.4. Pharmacokinetics

**Figure 2:**
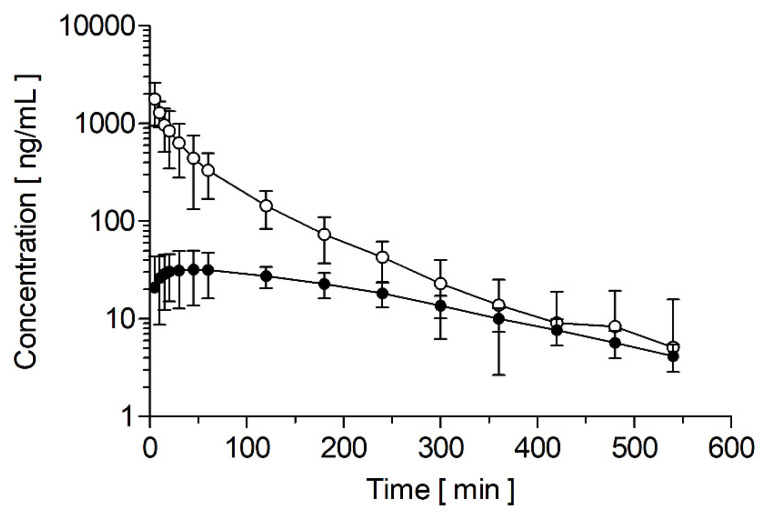
Plasma concentration-time profiles after a single fast intravenous bolus injection (within 1 min) of 1 mg/kg doxapram in German Landrace pigs (*n* = 3). Doxapram concentrations are depicted with white circles and 2-ketodoxapram with black circles. Data points are given as mean with 95% confidence interval.

After intravenous administration of 1 mg/kg doxapram, the three German landrace pigs showed biphasic pharmacokinetics (Figure 2). Very similar profiles have already been described in healthy humans [28,29], lambs [30], rabbits [21], and horses [26]. Furthermore, Robson and Prescott [28], and Clements et al. [29] were able to observe a third phase with an even lower rate of elimination, starting approximately 12 h after injection and, therefore, outside the observation window of this experiment. With an extension of the observation period, it would be possible to assess whether porcine pharmacokinetics have a third compartment. However, the contribution of a third compartment is considered negligible for doxapram exposure because the extrapolated fraction of the AUC_∞_ is only 0.66 ± 0.42% for doxapram and 8.60 ± 0.26% for 2-ketodoxapram. In the rabbit study, the sampling period was 24 h, but no details are given on the usage of all samples for pharmacokinetics [21]; in the lamb study, the sample period was 24 h, but only the first 6 h were used for the pharmacokinetic profile [30], and in the horse study, the sampling period was only 8 h [26]. Therefore, in all these animal studies, the sampling period was probably too short to assess the existence of a third phase in the pharmacokinetics of doxapram in these species. 

Table 2 summarizes the pharmacokinetic parameters after intravenous doxapram bolus injection; however, because the number of treated pigs is low (*n* = 3), the data should only be seen as preliminary. The elimination half-life (t_1/2_) of doxapram in pigs was 1.38 ± 0.22 h. Compared to other species, this value is between rabbits (0.47 ± 0.17 h) [21], lambs (5.2 h, range 1.2–11.6 h) [30], horses (2.62–3.29 h) [26], and humans (3.4 ± 0.7 h) [28]. Clements et al. [29] fitted the data from six healthy human volunteers to a three-compartmental model and observed an intermediate t_1/2_ of 1.03 ± 0.16 h and a terminal t_1/2_ of 5.92 ± 1.37 h. 

The maximal plasma concentrations (c_max_) of 1 mg/kg doxapram in pigs after intravenous bolus injection (within 1 min) was 1780 ± 275 ng/mL and was consistent with data from different species. After injection of 1.5 mg/kg doxapram (within 2–3 min) in healthy humans, a c_max_ of 2.6 µg/mL [29] and approximately 3 µg/mL [28] were measured. In lambs, after injection of 2.5 mg/kg doxapram (within 1 min), a c_max_ of 3.1 µg/mL (range: 2.7–3.6 µg/mL) was observed [30]. Similar values were observed in horses (1341 ± 256 ng/mL; 1.1 mg/kg doxapram; no injection time given) [26] and rabbits (1515 ± 130 ng/mL; 5 mg/kg doxapram; injection within 1 min) [21]. 

Furthermore, the clearance in pigs was 14.3 ± 2.0 mL/min/kg. In humans, clearances of 5.2 ± 1.7 mL/min/kg and 5.9 ± 1.0 mL/min/kg were observed [28,29]. In other species, a clearance of 11.1 ± 2.4 mL/min/kg (1.1 mg/kg doxapram) was reported for horses [26] and of 9.0 mL/min/kg (range: 5.7–13.3 mL/min/kg) for lambs [30]. The AUC in pigs (1186 ± 170 ng/mL*h) is similar to the AUC in horses after intravenous administration of 1.1 mg/kg doxapram (1728 ± 400 ng/mL*h) [26]. The AUC is higher in humans (4533 ± 1683 ng/mL*h; 1.5 mg/kg doxapram) [28] and lower in rabbits (2094 ± 100 ng/mL*h; 5 mg/kg doxapram) [21], considering the differences in administered dose.

To our knowledge, there are no data available on the pharmacokinetics of 2-ketodoxapram after intravenous administration of doxapram in any species. However, Bairam et al. [30] administered 2.5 mg/kg 2-ketodoxapram in new-born lambs and observed a t_1/2_ of 2.26 h (0.7–3.4 h), which is very similar to the value determined in pigs after administration of 1 mg/kg doxapram (2.42 ± 0.04 h). These data need to be interpreted carefully as they are based on only three pigs and after administration of doxapram and not 2-ketodoxapram; therefore, the pharmacokinetics of 2-ketodoxapram after its administration are expected to be different. 

### 3.5. Protein Binding

Both doxapram (95.5 ± 0.9%) and 2-ketodoxapram (98.4 ± 0.3%) are predominantly bound to proteins in porcine blood with a very small free fraction. The measurement was performed in samples taken 60 min after administration of 1 mg/kg doxapram, and the total concentration of doxapram was 332 ± 53.6 ng/mL and 31.7 ± 5.1 ng/mL of 2-ketodoxapram. In the literature, there are no data available on the protein binding of doxapram or 2-ketodoxapram in pigs. However, Sam et al. [26] measured the bound fraction of doxapram in horses with values between 76.0–85.4%, but they did not assess 2-ketodoxapram. Furthermore, they found that the protein binding is concentration dependent with an increase in free fraction with higher doxapram concentrations [26]. The considerably higher free fractions in horses could be caused by species-specific differences in protein binding as was already shown for other drugs [31]. The binding sites of plasma proteins vary between species, leading to different binding affinities [32]. For the porcine samples, no concentration dependence of the protein binding could be observed during the measurement of samples with different concentrations (data not shown). This could be connected to the before-mentioned species-specific differences in plasma protein configuration. The available protein binding sites in horses could be saturated at a lower doxapram concentration, leading to the observed non-linear characteristic, which seems to be absent in pigs in the same concentration range. Lastly, the use of different techniques in the determination of the free fraction could lead to the observed discrepancy. 

### 3.6. Brain-to-Plasma Ratio

**Figure 3:**
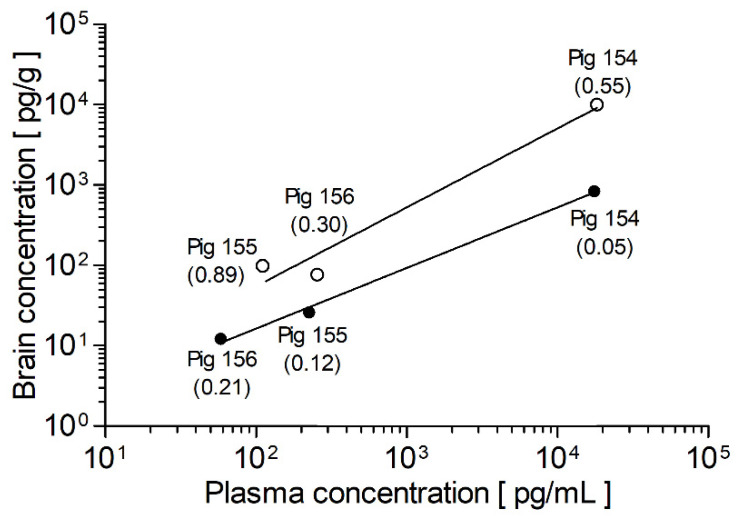
Comparison of plasma and brain tissue concentrations in German Landrace pigs (*n* = 3) after daily intravenous administration of 1 mg/kg doxapram at the end of a 14-day period. Brain-to-plasma ratio is given in parentheses. The values were fitted with a linear regression model with a slope of 0.98 ± 0.20 for doxapram and 0.75 ± 0.04 for 2-ketodoxapram. In this plot, higher brain-to-plasma ratios correlate with steeper slopes. Therefore, the steeper slope of doxapram indicates a higher brain-to-plasma ratio compared to 2-ketodoxapram. Doxapram values are depicted with white circles and 2-ketodoxapram with black circles.

For comparison of the brain tissue samples, the measurement result of each sample was divided by its individual weight, revealing much lower concentrations of doxapram and 2-ketdoxapram in the brain tissue compared to plasma samples taken at the time of the brain sampling (Figure 3). This observation is supported by data from Kumita et al. [33], who compared doxapram serum to cerebrospinal fluid (CSF) concentrations in premature infants and found higher concentrations in the serum than the CSF (CSF–serum ratio: 0.48 ± 0.13), and by data from Bruce et al. [34] who reported very low doxapram concentrations in the CSF of dogs. Furthermore, in pigs, the brain-to-plasma ratio observed for 2-ketodoxapram (0.12 ± 0.07) is 5.0-fold lower than the one for doxapram (0.58 ± 0.24). This result is unexpected because the lipophilic metabolite 2-ketodoxapram should be able to cross the blood–brain barrier more easily via passive diffusion than the more hydrophilic doxapram. Possible explanations are that 2-ketodoxapram is a substrate for efflux transporters, as are many small lipophilic compounds [35], or the almost three times higher free fraction of doxapram compared to 2-ketodoxapram, leading to a higher availability of unbound doxapram in the blood and, therefore, at the blood–brain barrier and possibly in the brain. However, due to the limited number of animals (*n* = 3) and the fact that pigs did not receive 2-keodoxapram directly, the results have to be interpreted carefully and should only be considered preliminary. 

## 4. Conclusions

We developed and validated a highly sensitive UPLC-MS/MS assay for the simultaneous quantification of doxapram and its active metabolite 2-ketodoxapram in porcine plasma, spanning at least six orders of magnitude (10 pg/mL–2500 ng/mL) with a LLOQ of 10 pg/mL. With this assay, it was also possible to accurately and precisely quantify doxapram and 2-ketodoxapram in brain tissue and to assess its free fraction in plasma. The assay was successfully applied to an animal pharmacokinetic study, which provided preliminary pharmacokinetic data in pigs. This first step is the basis for studies that evaluate the pharmacokinetic and pharmacodynamic relationship of doxapram and its metabolite in pigs and, after a transfer of the assay, in humans or other species. 

## Figures and Tables

**Table 1:** Overview of published quantification methods for doxapram and 2-ketodoxapram.

Study	Instruments	Range [ng/mL]	Analytes	Sample Matrix	Sample Volume	Run Time	Internal Standard	Sample Preparation
Aranda et al. (1988) [17]	HPLC-UV	1000–15,000	Doxapram, ketodoxapram, AHR 5904, AHR 0914	Human serum	50 µL	15 min	Beta-hydroxy-phenyl-theophylline	LLE
Barbé et al. (1999) [18]	HPLC-UV/VIS	100–20,000	Doxapram, ketodoxapram, AHR 5904, AHR 0914	Human plasma	60 µL	10 min	Butobarbital	LLE
Coutts et al. (1991) [19]	GC-N/P-D	- ^1^	Doxapram and many metabolites	Human urine	0.5–1 mL	- ^2^	-	LLE
Flint et al. (2018) [15]	UPLC-MS/MS	50–4500	Doxapram	Human plasma	50 µL	5 min	Fentanyl-d5	PP
		50–5000	Ketodoxapram					
Komatsu et al. (2005) [16]	GC-MS	250–5000	Doxapram	Human plasma	- ^2^	- ^2^	Diazepam	SPE
LeGatt et al. (1986) [20]	GC-N/P-D	100–10,000	Doxapram and ketodoxapram	Human plasma	200 µL	6 min ^3^	AHR-755 (doxapram analog)	LLE
Lin et al. (2011) [21]	LC-MS/MS	2–1000	Doxapram	Rabbit plasma	100 µL	10 min	Urapidil	PP
Nichol et al. (1980) [22]	GC-MS	-^2^	Doxapram	Human blood, plasma	50–100 µL	- ^2^	Dextromoramide	LLE
	GC-FID			Human urine	0.5–2 mL			
Ogawa et al. (2015) [23]	HPLC-UV/VIS	30–? ^4^	Doxapram	Human serum	50 µL	25 min ^3^	Butobarbital	LLE
		10–? ^4^	Ketodoxapram					
Robson and Prescott (1977) [24]	GC-N-D	10–5000	Doxapram	Human plasma	2 mL	- ^2^	Naftidrofuryl oxalte	LLE
		250–5000	Ketodoxapram					
Roozekrans et al. (2017) [25]	LC-MS/MS	2–5000	Doxapram	Human plasma	- ^2^	- ^2^	- ^2^	PP
Sams et al. (1992) [26]	GC-N/P-D	25–5000	Doxapram	Horse plasma	1 mL	- ^2^	Diazepam	LLE
Suzuki et al. (2017) [14]	LC-MS/MS	20–5000	Doxapram and ketodoxapram	Human serum	25 µL	17 min	Propranolol	PP
Torok-Both et al. (1985) [27]	GC-N/P-D	- ^2^	Doxapram	Human plasma	20–100 µL	8 min ^3^	Diazepam	PP and LLE
				Human urine	2–5 µL			

^1^ no quantification; ^2^ no information available; ^3^ run time approximated from description in manuscript; ^4^ unknown upper limit; FID: flame ionization detector; GC: gas chromatography; HPLC: high performance liquid chromatography; LC: liquid chromatography; LLE: liquid–liquid extraction; MS: mass spectrometer; MS/MS: tandem mass spectrometer; N-D: nitrogen detector; N/P-D: nitrogen–phosphorus detector; PP: protein precipitation; UPLC: ultra performance liquid chromatography; UV: ultraviolet detector; UV/VIS: ultraviolet/visible spectrum detector.

**Table 2:** Calculated pharmacokinetic parameters after a single intravenous bolus injection of 1 mg/kg doxapram in German Landrace Pigs (*n* = 3). Mean data are shown ± SD.

Animal	C_max_ [ng/mL]	AUC [ng/mL*h]	V_SS_ [L]	Cl [mL/min/kg]	t_1/2_ [h]	t_max_ [h]
Doxapram						
Pig 154	2149	1169	34.4	14.2	1.18	-
Pig 155	1490	986	51.5	16.9	1.26	-
Pig 156	1701	1403	37.1	11.9	1.69	-
Mean	1780 ± 275	1186 ± 170	41.0 ± 7.5	14.3 ± 2.0	1.38 ± 0.22	-
2-Ketodoxapram						
Pig 154	31.2	157	-	-	2.47	0.33
Pig 155	26.2	139	-	-	2.36	1.00
Pig 156	39.5	185	-	-	2.42	0.75
Mean	32.3 ± 5.5	160 ± 19	-	-	2.42 ± 0.04	0.69 ± 0.28

AUC: area under the concentration-time curve extrapolated to infinity; Cl: clearance; c_max_: maximal plasma concentration; t_1/2_: elimination half-life; t_max_: time to reach maximal plasma concentration; V_ss_: volume of distribution.

## Data Availability

The data are available from the corresponding author upon reasonable request.

## Supplementary Materials

The following supporting information can be downloaded at: https://www.mdpi.com/article/10.3390/pharmaceutics14040762/s1, Figure S1: Tandem mass spectrum (MS/MS) of doxapram and 2-ketodoxapram; Figure S2: Selected chromatograms of the assay validated for quantification of the high plasma concentrations; Figure S3: Selected chromatograms of the assay validated for the quantification of the low plasma concentrations; Figure S4: Selected chromatograms of the assay validated for the quantification of brain tissue concentrations; Table S1: Matrix effect and recovery data of the assay validated for quantification of high plasma concentrations; Table S2: Matrix effect and recovery data of the assay validated for the quantification of low plasma concentrations; Table S3: Matrix effect and recovery data of the assay validated for the quantification of brain tissue; Table S4: Accuracy and precision data of the assay validated for the quantification of high plasma concentrations; Table S5: Accuracy and precision data of the assay validated for the quantification of low plasma concentrations; Table S6: Accuracy and precision data of the assay validated for the quantification of brain tissue; Table S7: Stability data for the assay validated for the quantification of high plasma concentrations; Table S8: Stability data for the assay validated for the quantification of low plasma concentrations; Table S9: Stability data for the assay validated for the quantification of brain tissue; Table S10: Incurred sample reanalysis data for the assay validated for the quantification of high plasma concentrations; Table S11: Incurred sample reanalysis data for the assay validated for the quantification of low plasma concentrations; Table S12: Incurred sample reanalysis data for the assay validated for the quantification of brain tissue.
