# Supplementary material for: Simultaneous Quantification and Pharmacokinetic Characterization of Doxapram and 2-Ketodoxapram in Porcine Plasma and Brain Tissue
Source: Pharmaceutics. 2022 Mar 31;14(4):762. doi: 10.3390/pharmaceutics14040762 (PMC9031635; doi:10.3390/pharmaceutics14040762)
Supplement: Supplementary file 1 [file pharmaceutics-14-00762-s001.zip › pharmaceutics-1638703-supplementary.pdf]

pharmaceutics

MDPI

Supplementary Materials: Simultaneous Quantification and Pharmacokinetic Characterization of Doxapram and 2-Ketodoxapram in Porcine Plasma and Brain Tissue

Manuel Kraft, Kathrin I. Foerster, Felix Wiedmann, Max Sauter, Amelie Paasche, Pablo L. Blochberger, Baran Yesilgöz, Yannick L'hoste, Norbert Frey, Walter E. Haefeli, Jürgen Burhenne and Constanze Schmidt

Supplementary Figures:

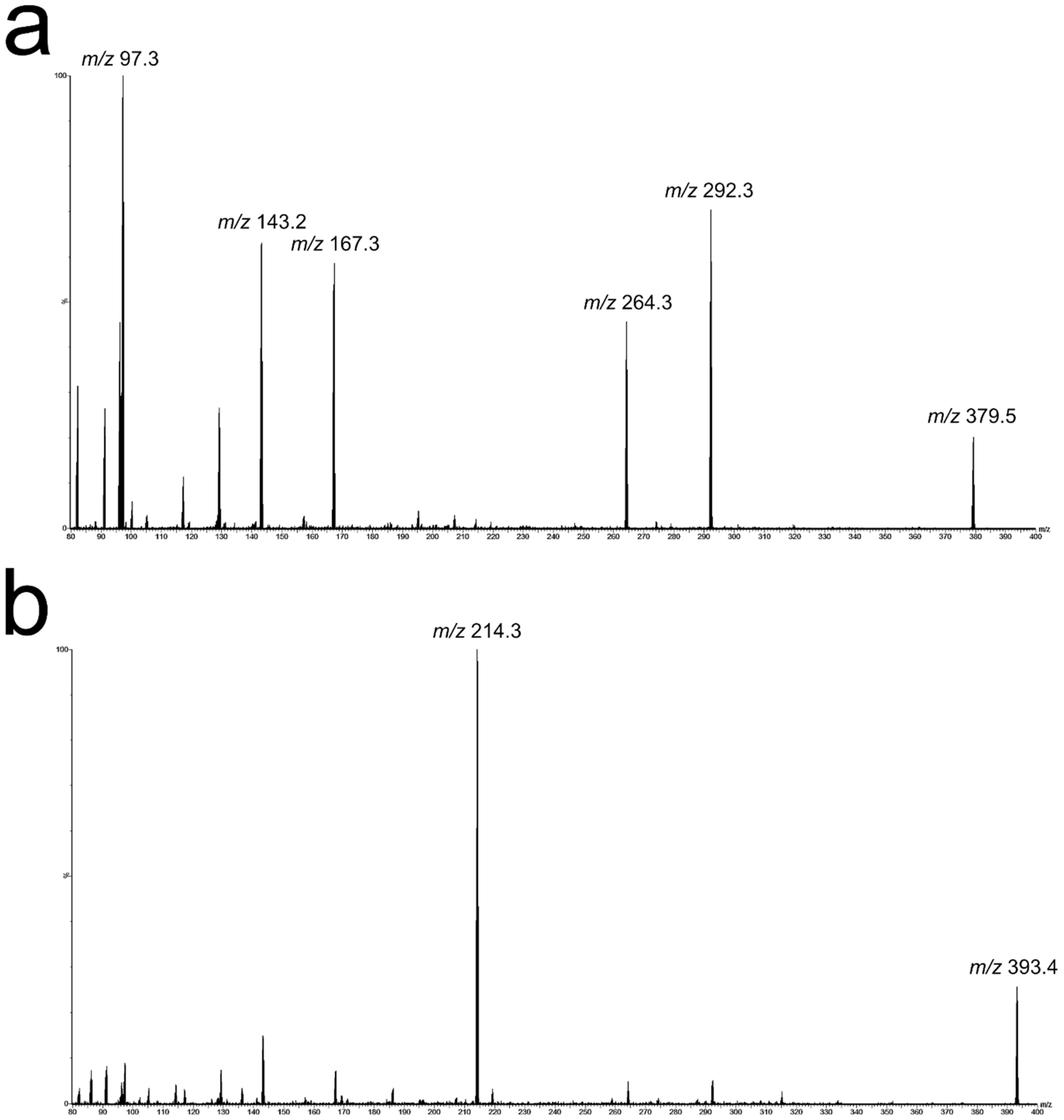

Figure S1. Tandem mass spectrum (MS/MS) of doxapram and 2-ketodoxapram. Tandem mass spectrum using electrospray ionization in positive mode of the $[M+H]^+$ signal *m/z* 379.5 (doxapram; (**a**) after collision-induced decomposition with a collision energy of 18 V and of the $[M+H]^+$ signal *m/z* 393.4 (2-ketodoxapram; (**b**) applying a collision energy of 23 V.

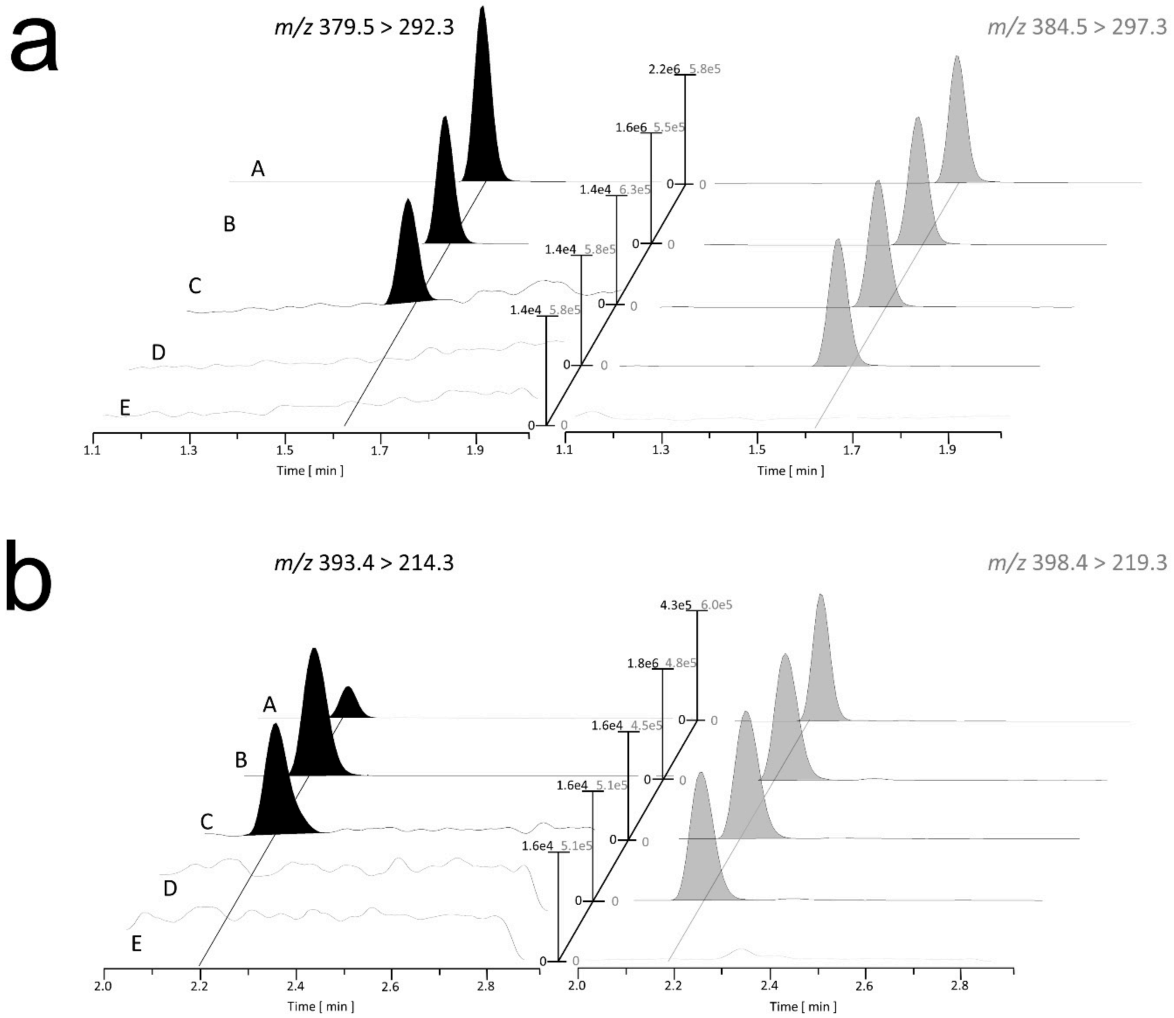

Figure S2. Selected chromatograms of the assay validated for the quantification of high plasma concentrations. Analyte transition is depicted on the left side in black and internal standard (IS) transition on the right side in grey. Top (**a**): transitions of doxapram and the IS doxapram-d5; Bottom (**b**): transitions of 2-ketodoxapram and the IS ketodoxapram-d5. (A) plasma sample taken 15 min after administration of 1 mg/kg doxapram to pig #154 (representing 1,095 ng/mL doxapram and 30.1 ng/mL 2-ketodoxapram), (B) sample at mid quality control (QC) concentration (150 ng/mL), (C) sample at lower limit of quantification (LLOQ) concentration (1 ng/mL), (D) sample with added IS, and (E) blank sample. Intensities of blank samples were normalized to the intensity of the LLOQ sample. The plasma sample was normalized to the intensity of the QC sample. The transition of IS and the analyte were processed separately and independently for doxapram and 2-ketodoxapram.

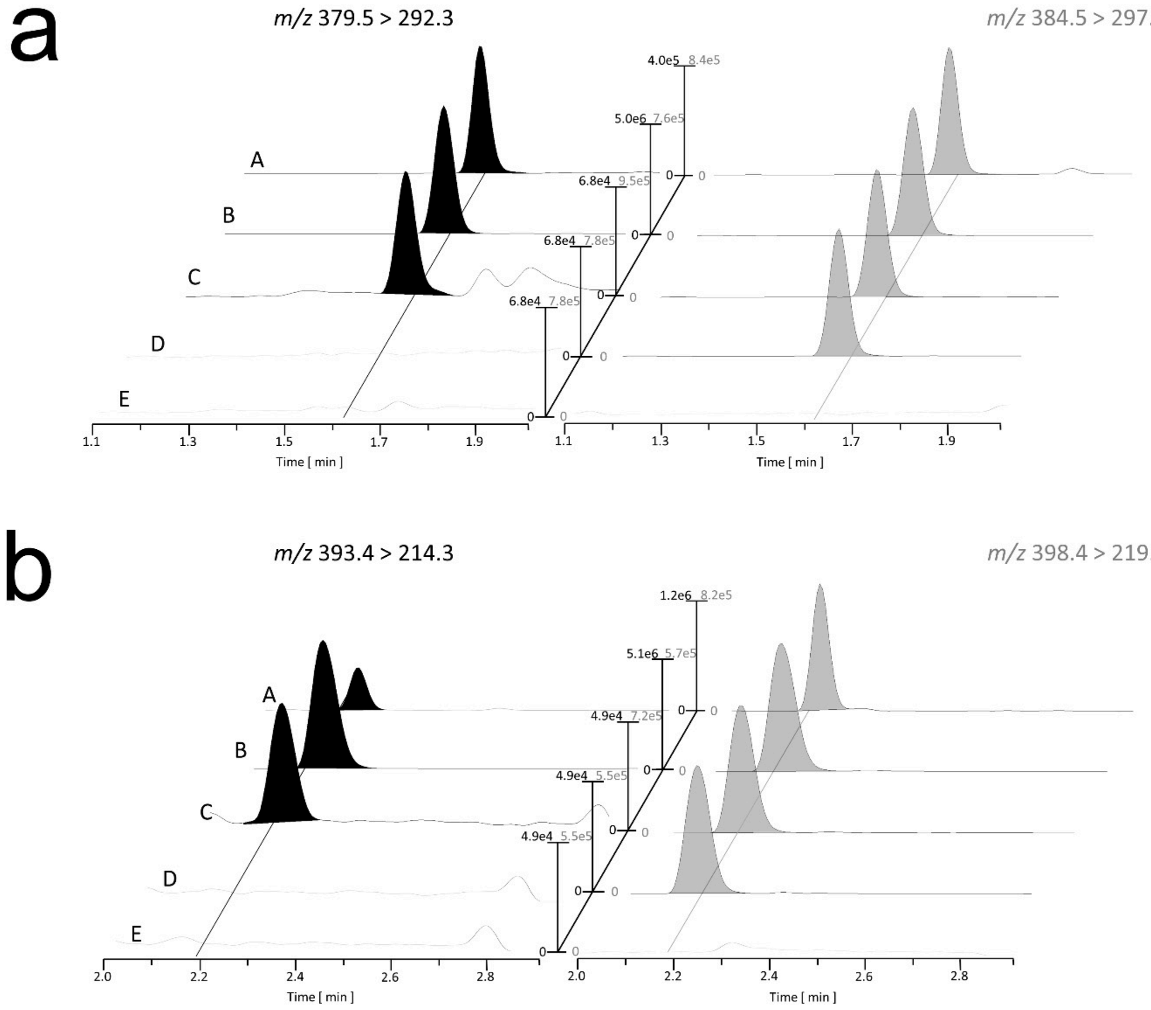

Figure S3. Selected chromatograms of the assay validated for quantification of low plasma concentrations. Analyte transition is depicted on the left side in black and internal standard (IS) transition on the right side in grey. Top (**a**): transitions of doxapram and the IS doxapram-d5; Bottom (**b**): transitions of 2-ketodoxapram and the IS ketodoxapram-d5. (A) plasma sample taken at the final operation after 14 d of daily administration of 1 mg/kg doxapram to pig #155 (representing 111 pg/mL doxapram and 225 pg/mL 2-ketodoxapram), (B) sample at mid quality control (QC) concentration (1,500 pg/mL), (C) sample at lower limit of quantification (LLOQ) concentration (10 pg/mL), (D) sample with added IS, and (E) blank sample. Intensities of blank samples were normalized to the intensity of the LLOQ sample. The plasma sample was normalized to the intensity of the QC sample. The transition of IS and the analyte were processed separately and independently for doxapram and 2-ketodoxapram.

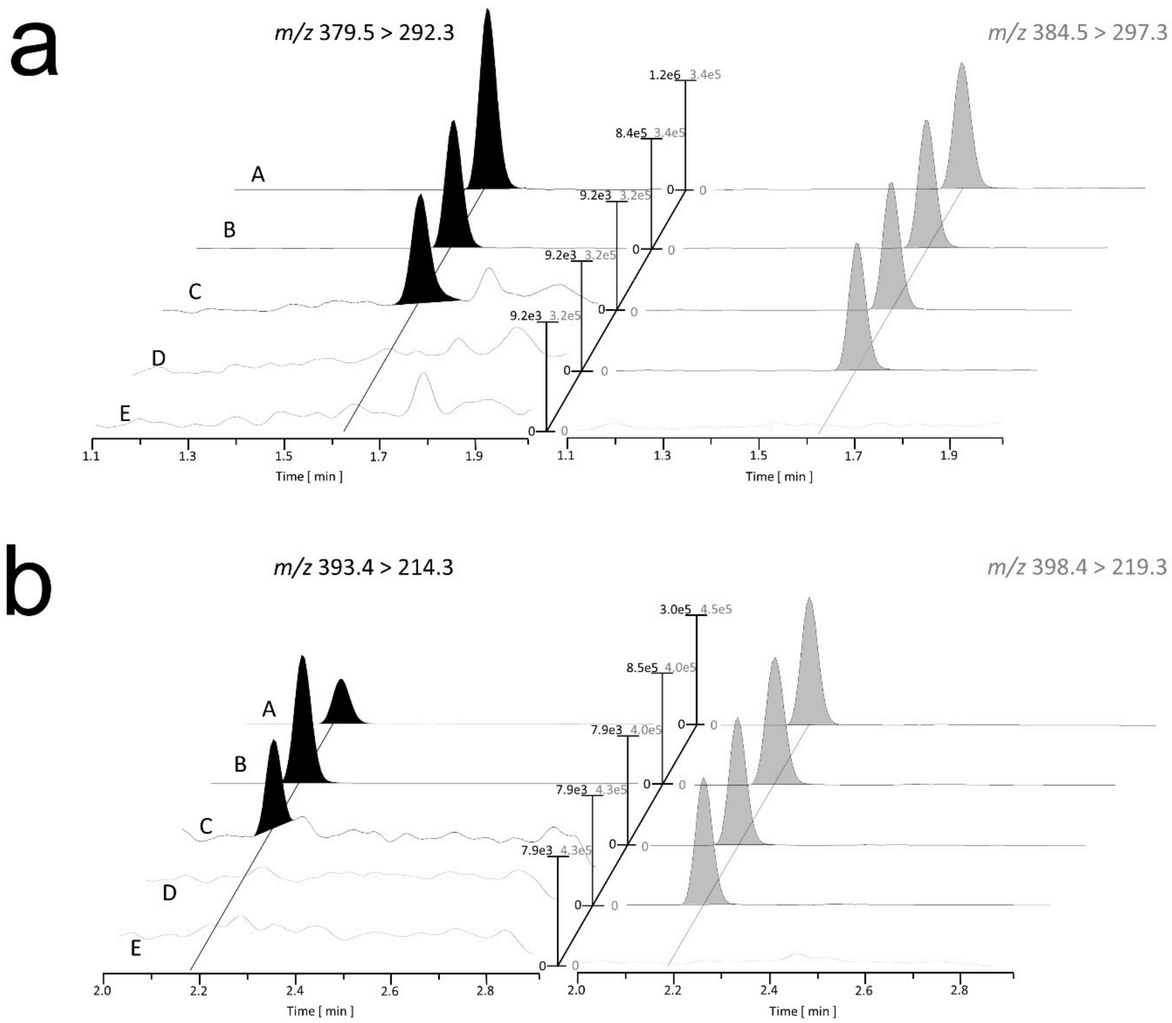

Figure S4. Selected chromatograms of the assay validated for quantification of brain tissue concentrations. Analyte transition is depicted on the right side in black and internal standard (IS) transition on the right side in grey. Top (**a**): transitions of doxapram and the IS doxapram-d5; Bottom (**b**): transitions of 2-ketodoxapram and the IS ketodoxapram-d5. (A) brain tissue sample taken at the final operation after 14 d of daily administration of 1 mg/kg doxapram to pig #154 (representing 164 pg/sample doxapram and 37.3 pg/sample 2-ketodoxapram), (B) sample at mid quality control (QC) concentration (150 pg/sample), (C) sample at lower limit of quantification (LLOQ) concentration (1 pg/sample), (D) sample with added IS, and (E) blank sample. Intensities of blank samples were normalized to the intensity of the LLOQ sample. The plasma sample was normalized to the intensity of the QC sample. The transition of IS and the analyte were processed separately and independently for doxapram and 2-ketodoxapram.

Supplementary Tables:

Table S1. Matrix effect and recovery data of the assay validated for quantification of high plasma concentrations.

High Plasma Concentrations	Doxapram				2-Ketodoxapram			
	QC A	QC B	QC C	QC D	QC A	QC B	QC C	QC D
Nominal value [ng/mL]	3	15	150	1,500	3	15	150	1,500
Matrix effect [%]								
Lot #1	95.0	99.7	100.9	99.5	96.9	99.4	101.1	100.1
Lot #2	98.8	99.4	99.7	100.0	103.6	99.4	100.0	100.4
Lot #3	95.0	99.7	100.5	99.8	101.8	99.3	100.1	100.1
Lot #4	100.6	99.6	100.8	99.5	101.8	99.8	100.0	100.4
Lot #5	98.8	100.5	100.3	99.9	100.0	99.0	100.3	100.2
Lot #6	96.3	101.0	100.8	99.3	100.4	99.4	99.4	99.7
Mean	97.4	100.0	100.5	99.7	100.8	99.3	100.1	100.2
Recovery [%]								
Lot #1	99.3	98.1	97.7	99.0	100.2	98.5	97.8	98.6
Lot #2	95.6	98.4	98.9	98.5	93.7	98.6	98.9	98.3
Lot #3	99.3	98.1	98.1	98.6	95.4	98.6	98.8	98.6
Lot #4	93.8	98.2	97.8	98.9	95.4	98.1	98.9	98.3
Lot #5	95.6	97.3	98.3	98.6	97.1	98.9	98.6	98.6
Lot #6	98.1	96.8	97.8	99.2	96.7	98.5	99.5	99.0
Mean	96.9	97.8	98.1	98.8	96.4	98.6	98.7	98.6

Internal standard (IS)-normalized matrix effect and recovery were evaluated in six different lots of blank matrix at all four QC levels. All values are within the required limit of ±15%.

Table S2. Matrix effect and recovery data of the assay validated for quantification of low plasma concentrations.

Low Plasma Concentrations	Doxapram				2-Ketodoxapram			
	QC A	QC B	QC C	QC D	QC A	QC B	QC C	QC D
Nominal value [pg/mL]	30	150	1,500	7,500	30	150	1,500	7,500
Matrix effect [%]								
Lot #1	113.4	101.8	100.4	99.7	97.6	99.0	98.3	98.3
Lot #2	112.9	104.4	100.4	99.2	99.6	99.9	98.1	98.7
Lot #3	105.5	100.7	100.7	99.2	99.1	99.2	98.5	99.0
Lot #4	106.5	101.3	100.3	99.2	99.3	99.0	98.4	98.7
Lot #5	111.0	103.5	99.6	99.9	98.9	98.6	98.4	98.6
Lot #6	113.4	101.3	101.0	98.9	99.7	99.2	98.9	99.2
Mean	110.4	102.1	100.4	99.4	98.9	99.2	98.4	98.7
Recovery [%]								
Lot #1	95.4	98.6	97.5	98.8	98.6	99.6	98.3	99.8
Lot #2	95.8	96.1	97.6	99.4	96.6	98.7	98.6	99.4
Lot #3	102.6	99.6	97.3	99.3	97.2	99.5	98.2	99.0
Lot #4	101.7	99.0	97.6	99.3	97.0	99.6	98.2	99.4
Lot #5	97.5	96.9	98.3	98.6	97.3	100.1	98.3	99.5
Lot #6	95.4	99.0	96.9	99.6	97.2	99.5	97.7	98.9
Mean	98.0	98.3	97.5	99.2	97.3	99.5	98.2	99.3

Internal standard (IS)-normalized matrix effect and recovery were evaluated in six different lots of blank matrix at all four QC levels. All values are within the required limit of ±15%.

Table S3. Matrix effect and recovery data of the assay validated for quantification of brain tissue.

Brain Tissue	Doxapram				2-Ketodoxapram			
	QC A	QC B	QC C	QC D	QC A	QC B	QC C	QC D
Nominal value [pg/XX]	1.5	15	150	1,500	1.5	15	150	1,500
Matrix effect [%]								
Lot #1	107.8	98.6	100.4	100.6	100	101.9	103.4	102.7
Lot #2	107.8	100.4	99.1	99.7	114.1	102.5	102.8	102.7
Lot #3	103.2	101.1	100.4	99.3	114.1	103.0	103.0	102.5
Lot #4	109.1	99.0	99.5	101.5	101.3	104.3	101.8	101.6
Lot #5	110.4	100.3	99.6	99.8	110.3	100.6	101.0	100.2
Lot #6	109.1	101.5	101.0	100.7	107.7	100.6	100.1	101.9
Mean	108.1	100.2	100.0	100.3	107.9	102.1	102.0	102.0
Recovery [%]								
Lot #1	91.6	103.0	100.6	98.2	100.0	98.6	99.1	98.6
Lot #2	91.6	101.2	101.9	99.1	87.6	98.0	99.7	98.6
Lot #3	95.6	100.5	100.6	99.5	87.6	97.5	99.5	98.8
Lot #4	90.5	102.6	101.5	97.3	98.7	96.4	100.7	99.6
Lot #5	89.4	101.3	101.4	99.0	90.7	99.9	101.6	101.1
Lot #6	90.5	100.1	100.0	98.1	92.9	99.6	102.4	99.4
Mean	91.0	101.4	101.0	98.5	92.7	98.4	100.5	99.3

Internal standard (IS)-normalized matrix effect and recovery were evaluated in six different lots of blank matrix at all four QC levels. All values are within the required limit of ±15%.

Table S4. Accuracy and precision data of the assay validated for quantification of high plasma concentrations.

High Plasma Concentrations		Doxapram					2-Ketodoxapram				
		LLOQ	QC A	QC B	QC C	QC D	LLOQ	QC A	QC B	QC C	QC D
		1	3	15	150	1,500	1	3	15	150	1,500
**Within run**											
1	Mean [ng/mL]	1.0	2.7	14.0	145.2	1,447	1.1	3.2	15.7	160.5	1,555
	Accuracy [%]	99.8	90.3	93.1	96.8	96.5	107.4	105.0	104.6	107.0	103.6
	Precision [%]	7.9	3.3	4.0	0.9	0.9	3.6	3.5	2.8	0.7	1.1
	correlation coefficient			0.999					0.999		
2	Mean [ng/mL]	1.0	2.8	13.9	144.2	1,435	1.0	3.1	15.6	159.2	1,566
	Accuracy [%]	104.3	93.3	92.9	96.1	95.7	103.1	102.3	104.0	106.1	104.4
	Precision [%]	5.4	3.3	1.0	1.1	1.1	7.6	3.8	0.6	0.6	0.9
	correlation coefficient			0.999					0.999		
3	Mean [ng/mL]	1.1	2.8	14.0	142.1	1,431	1.1	3.1	15.6	158.3	1,562
	Accuracy [%]	107.7	91.9	93.4	94.7	95.4	108.1	103.9	103.7	105.5	104.1
	Precision [%]	4.0	2.8	2.9	2.3	3.0	6.4	4.2	2.1	1.5	3.1
	correlation coefficient			0.999					0.999		
Between runs											
	Mean [ng/mL]	1.0	2.8	14.0	143.8	1,438	1.1	3.1	15.6	159.3	1,561
	Accuracy [%]	103.7	91.8	93.1	95.9	95.8	106.1	103.7	104.1	106.2	104.0
	Precision [%]	6.5	3.3	2.7	1.7	1.9	6.0	3.8	2.0	1.1	1.9

Within run (intra-day) and between runs (inter-day) comparisons were evaluated six-fold in three separate batches at all four QC levels. All values are within the required limit of ±15%.

Table S5. Accuracy and precision data of the assay validated for quantification of low plasma concentrations.

**Low Plasma Concentrations**		Doxapram					2-Ketodoxapram				
		LLOQ	QC A	QC B	QC C	QC D	LLOQ	QC A	QC B	QC C	QC D
		10	30	150	1,500	7,500	10	30	150	1,500	7,500
**Within run**											
1	Mean [pg/mL]	10.7	28.4	143	1,437	7,157	10.6	31.6	158	1,580	7,864
	Accuracy [%]	106.8	94.8	95.2	95.8	95.4	106.4	105.2	105.5	105.3	104.9
	Precision [%]	3.6	5.5	0.9	1.3	0.4	4.3	1.0	1.4	0.9	0.7
	correlation coefficient			0.999					0.999		
2	Mean [pg/mL]	10.0	27.0	142	1,419	6,939	10.6	31.7	157	1,568	7,712
	Accuracy [%]	99.5	90.0	94.5	94.6	92.5	105.8	105.8	104.6	104.6	102.8
	Precision [%]	2.4	2.5	0.5	1.5	1.0	3.4	2.2	1.5	1.6	0.9
	correlation coefficient			0.999					0.999		
3	Mean [pg/mL]	10.6	27.5	142	1,442	7,192	10.1	31.1	158	1,589	7,802
	Accuracy [%]	106.1	91.5	94.5	96.1	95.9	100.6	103.6	105.5	105.9	104.0
	Precision [%]	6.4	3.0	1.8	0.7	2.6	3.8	1.7	0.6	0.8	4.8
	correlation coefficient			0.999					0.999		
Between runs											
	Mean [pg/mL]	10.4	27.6	142	1,433	7,096	10.5	31.5	158	1,579	7,793
	Accuracy [%]	104.0	91.9	94.7	95.5	94.6	104.5	104.9	105.2	105.3	103.9
	Precision [%]	5.3	4.2	1.2	1.3	2.3	4.4	1.8	1.2	1.2	2.8

Within run (intra-day) and between runs (inter-day) comparisons were evaluated six-fold in three separate batches at all four QC levels. All values are within the required limit of ±15%.

Table S6. Accuracy and precision data of the assay validated for quantification of brain tissue.

	Brain Tissue	Doxapram					2-Ketodoxapram				
		LLOQ	QC A	QC B	QC C	QC D	LLOQ	QC A	QC B	QC C	QC D
		1	1.5	15	150	1,500	1	1.5	15	150	1,500
	**Within run**										
1	Mean [pg/sample]	1.0	1.4	14.1	148	1,496	1.1	1.6	16.3	163	1,652
	Accuracy [%]	96.4	93.8	93.7	98.6	99.7	107.5	105.8	108.4	108.7	110.1
	Precision [%]	5.6	3.2	3.9	1.2	1.8	4.7	2.2	1.3	0.7	0.9
	correlation coefficient			0.998					0.998		
2	Mean [pg/sample]	1.0	1.6	14.5	149	1,473	1.0	1.6	15.7	166	1,649
	Accuracy [%]	101.6	106.6	96.7	99.1	98.2	98.8	105.5	104.8	110.7	109.9
	Precision [%]	7.6	3.7	1.9	0.9	0.9	8.4	3.9	1.3	1.0	0.8
	correlation coefficient			0.999					0.999		
3	Mean [pg/sample]	1.0	1.5	15.2	152	1,544	1.0	1.4	15.8	161	1,603
	Accuracy [%]	101.6	99.6	101.4	101.5	102.9	98.1	95.3	105.4	107.2	106.9
	Precision [%]	6.0	8.0	2.4	2.2	0.8	8.3	6.3	2.9	1.7	2.2
	correlation coefficient			0.999					0.999		
	Between runs										
	Mean [pg/sample]	1.0	1.5	14.6	150	1,504	1.0	1.5	15.9	163	1,635
	Accuracy [%]	99.8	100.0	97.3	99.7	100.3	101.6	102.6	106.2	108.9	109.0
	Precision [%]	6.6	7.4	4.3	2.0	2.3	8.0	6.2	2.4	1.8	1.9

Within run (intra-day) and between runs (inter-day) comparisons were evaluated six-fold in three separate batches at all four QC levels. All values are within the required limit of ±15%.

Table S7. Stability data for the assay validated for quantification of high plasma concentrations.

High Plasma Concentrations	Doxapram				2-Ketodoxapram			
	**QC A**	**QC B**	**QC C**	**QC D**	**QC A**	**QC B**	**QC C**	**QC D**
	**3**	**15**	**150**	**1,500**	**3**	**15**	**150**	**1,500**
Freeze-and-thaw								
Mean [ng/mL]	2.8	13.98	143.2	1,421	3.1	15.7	158.5	1,546
Accuracy [%]	92.9	93.2	95.5	94.8	101.7	104.4	105.6	103.1
Precision [%]	1.9	0.6	0.7	1.0	2.3	1.4	0.5	0.5
Autosampler								
Mean [ng/mL]	2.8	13.7	141.1	1,410	3.1	15.3	157.3	1,538
Accuracy [%]	93.1	91.1	94.1	94.0	103.3	101.9	104.8	102.5
Precision [%]	1.6	5.6	0.6	0.6	5.3	4.8	0.6	0.9
Stability -20 °C								
Mean [ng/mL]	2.8	14.3	146.1	1,421	3.1	16.1	160.2	1,534
Accuracy [%]	94.1	95.1	97.4	94.7	103.7	107.3	106.8	102.2
Precision [%]	3.3	2.8	1.6	0.6	2.3	2.6	0.9	0.8
Stability RT								
Mean [ng/mL]	2.9	14.2	145.4	1,447	3.1	15.9	162.3	1,562
Accuracy [%]	96.1	94.7	96.9	96.5	104.2	105.9	108.2	104.2
Precision [%]	1.1	6.2	0.9	2.3	0.6	2.0	1.5	1.8

Accuracy and precision values after three freeze-and-thaw cycles, storing overnight in the autosampler at 15 °C, after 16 d in the freezer at -20 °C, or after 24 h at room temperature (RT). All measurements have been conducted in triplicates.

Table S8. Stability data for the assay validated for quantification of low plasma concentrations.

Low Plasma Concentrations	Doxapram				2-Ketodoxapram			
	**QC A**	**QC B**	**QC C**	**QC D**	**QC A**	**QC B**	**QC C**	**QC D**
	**30**	**150**	**1,500**	**7,500**	**30**	**150**	**1,500**	**7,500**
Freeze-and-thaw								
Mean [pg/mL]	27.2	140.2	1,446.2	7,092	31.1	156.0	1,581.4	7,775
Accuracy [%]	90.8	93.4	96.4	94.6	103.7	104.0	105.4	103.7
Precision [%]	7.7	1.3	1.0	1.5	1.7	1.0	0.4	1.9
Autosampler								
Mean [pg/mL]	26.7	138.9	1,414.1	6,997	32.7	157.3	1,568.3	7,868
Accuracy [%]	89.0	92.6	94.3	93.3	109.1	104.9	104.6	104.9
Precision [%]	3.6	1.4	1.0	0.9	2.0	0.7	0.9	0.4
Stability -20 °C								
Mean [pg/mL]	26.7	142.9	1,424.7	7,123	31.1	161.0	1,579.8	7,769
Accuracy [%]	89.1	95.3	95.0	95.0	103.6	107.3	105.3	103.6
Precision [%]	1.6	1.2	2.6	0.2	0.4	0.6	1.9	0.9
Stability RT								
Mean [pg/mL]	27.1	142.3	1,431.5	7,088	30.8	157.1	1,565.9	7,736
Accuracy [%]	90.3	94.8	95.4	94.5	102.7	104.7	104.4	103.2
Precision [%]	3.0	1.8	1.0	0.5	1.3	0.5	1.2	0.8

Accuracy and precision values after three freeze-and-thaw cycles, after storing overnight in the autosampler at 15 °C, after 16 d in the freezer at -20 °C, or after 24 h at room temperature (RT). All measurements have been conducted in triplicates.

Table S9. Stability data for the assay validated for quantification of brain tissue.

Brain Tissue	Doxapram				2-Ketodoxapram			
	**QC A**	**QC B**	**QC C**	**QC D**	**QC A**	**QC B**	**QC C**	**QC D**
	**1.5**	**15**	**150**	**1,500**	**1.5**	**15**	**150**	**1,500**
Freeze-and-thaw								
Mean [pg/sample]	1.4	14.1	146.7	1,522	1.7	16.6	167.8	1,687
Accuracy [%]	91.2	93.9	97.8	101.5	110.9	110.6	111.9	112.5
Precision [%]	2.4	3.9	2.7	2.4	2.3	0.9	2.1	1.0
Autosampler								
Mean [pg/sample]	1.5	14.7	151.5	1,511	1.4	16.0	158.8	1,544
Accuracy [%]	98.9	98.2	101.0	100.7	94.3	106.6	105.9	102.9
Precision [%]	4.4	2.5	0.4	0.9	4.3	4.7	1.4	1.0

Accuracy and precision values after three freeze-and-thaw cycles or storing overnight in the autosampler at 15 °C. All measurements have been conducted in triplicates.

Table S10. Incurred sample reanalysis data for the assay validated for quantification of high plasma concentrations.

High Plasma Sample	Doxapram			2-Ketodoxapram		
	Reanalysis [ng/mL]	Original [ng/mL]	Deviation [%]	Reanalysis [ng/mL]	Original [ng/mL]	Deviation [%]
A250DP154A04P	2,032	2,149	5.6	18.7	20.9	11.1
A250DP154A05P	1,426	1,423	-0.2	25.8	25.9	0.4
A250DP154A06P	1,095	1,088	-0.6	30.1	30.7	2.2
A250DP154A07P	858	893	4.0	30.1	31.2	3.4
A250DP154A08P	610	619	1.5	30.5	30.9	1.4
A250DP154A09P	408	413	1.4	29.2	31.2	6.8
A250DP154A10P	310	321	3.3	30.1	30.5	1.6
A250DP154A11P	130	129	-1.1	27.6	27.5	-0.2
A250DP154A12P	59.8	61.8	3.3	21.9	21.9	-0.2
A250DP154A13P	33.8	34.9	3.2	17.3	17.4	0.8
A250DP154A14P	19.1	19.0	-0.7	13.1	13.1	-0.1
A250DP154A15P	10.5	11.0	4.1	9.39	9.42	0.3
A250DP154A16P	6.94	7.04	1.4	7.22	7.29	0.9
A250DP156A04P	1,648	1,702	3.2	29.2	30.0	2.6
A250DP156A05P	1,013	1,343	28.0	26.0	33.3	24.8
A250DP156A06P	1,026	1,052	2.5	33.9	34.3	1.2
A250DP156A07P	935	1,003	7.0	33.7	36.3	7.5
A250DP156A08P	684	784	13.6	32.8	38.7	16.6
A250DP156A09P	508	578	12.9	35.1	39.5	11.8
A250DP156A10P	363	402	10.2	34.0	38.5	12.3
A250DP156A11P	164	172	4.7	29.0	29.9	3.0
A250DP156A12P	84.8	89.9	5.8	23.2	25.7	10.2
A250DP156A13P	46.2	50.3	8.4	19.1	20.6	7.8
A250DP156A14P	29.3	30.9	5.1	14.7	15.3	4.0
A250DP156A15P	17.8	19.1	7.1	10.6	11.4	7.0
A250DP156A16P	12.2	13.7	11.6	8.17	8.73	6.6
A250DP156A17P	11.6	12.7	9.7	5.89	6.50	9.9
A250DP156A18P	9.00	10.1	11.2	4.29	4.73	9.7

Of the tested 28 samples, 96.4% (27 samples) were within the limit of ±20% for doxapram as well as 2-ketodoxapram.

Table S11. Incurred sample reanalysis data for the assay validated for quantification of low plasma concentrations.

Low Plasma Sample	Doxapram			2-Ketodoxapram		
	Reanalysis [pg/mL]	Original [pg/mL]	Deviation [%]	Reanalysis [pg/mL]	Original [pg/mL]	Deviation [%]
A250DP154A19P	192	193	0.2	86.7	83.7	-3.6
A250DP154A41P	24,608	6,830	-113.1	471	473	0.4
A250DP154A58P	640	644	0.6	365	342	-6.6
A250DP155A19P	345	340	-1.6	99.9	94.5	-5.5
A250DP155A20P	1,243	1,157	-7.2	217	195	-10.5
A250DP155A38P	260	273	5.0	174	178	2.0
A250DP155A39P	2,776	2,763	-0.5	224	211	-6.0
A250DP155A40P	8,242	8,872	7.4	313	340	8.4
A250DP155A57P	6,036	5,560	-8.2	309	283	-8.8
A250DP155A75P	102	111	8.0	271	225	-18.7
A250DP156A23P	407	437	7.2	118	106	-10.7
A250DP156A24P	8,436	8,029	-4.9	87.2	79.7	-9.0
A250DP156A42P	9,978	9,128	-8.9	59.1	52.6	-11.6
A250DP156A60P	159	254	46.0	55.8	58.3	4.3

Of the tested 14 samples, 85.7% (12 samples) were within the limit of ±20% for doxapram. After excluding one sample outside the calibration range for doxapram, 92.3% of the samples were within the limit. All 14 2-ketodoxapram samples were inside the limit.

Table S12. Incurred sample reanalysis data for the assay validated for quantification of brain tissue.

Brain Tissue Sample	Doxapram			2-Ketodoxapram		
	Reanalysis [pg/sample]	Original [pg/sample]	Deviation [%]	Reanalysis [pg/sample]	Original [pg/sample]	Deviation [%]
P154A1	41.7	37.1	-11.7	2.56	2.24	-13.6
P154B1	75.2	69.8	-7.5	3.03	2.75	-9.5

Both analysed samples were within the limit of ±20% for doxapram as well as 2-ketodoxapram.
